# Dietary *Enteromorpha* Polysaccharide Enhances Intestinal Immune Response, Integrity, and Caecal Microbial Activity of Broiler Chickens

**DOI:** 10.3389/fnut.2021.783819

**Published:** 2021-11-29

**Authors:** Teketay Wassie, Zhuang Lu, Xinyi Duan, Chunyan Xie, Kefyalew Gebeyew, Zhang Yumei, Yulong Yin, Xin Wu

**Affiliations:** ^1^Key Laboratory of Agro-ecological Processes in Subtropical Region, National Engineering Laboratory for Pollution Control and Waste Utilization in Livestock and Poultry Production, Hunan Provincial Engineering Research Center for Healthy Livestock and Poultry Production, Institute of Subtropical Agriculture, Chinese Academy of Sciences, Changsha, China; ^2^College of Resources and Environment, Hunan Agricultural University, Changsha, China; ^3^Tianjin Institute of Industrial Biotechnology, Chinese Academy of Sciences, Tianjin, China

**Keywords:** chicken, *Enteromorpha* polysaccharide, immunity, intestinal integrity, marine algae, microbiota

## Abstract

Marine algae polysaccharides have been shown to regulate various biological activities, such as immune modulation, antioxidant, antidiabetic, and hypolipidemic. However, litter is known about the interaction of these polysaccharides with the gut microbiota. This study aimed to evaluate the effects of marine algae *Enteromorpha (Ulva) prolifera* polysaccharide (EP) supplementation on growth performance, immune response, and caecal microbiota of broiler chickens. A total of 200 1-day-old Ross-308 broiler chickens were randomly divided into two treatment groups with ten replications of ten chickens in each replication. The dietary treatments consisted of the control group (fed basal diet), and EP group (received diet supplemented with 400 mg EP/kg diet). Results showed that chickens fed EP exhibited significantly higher (*P* < 0.05) body weight and average daily gain than the chicken-fed basal diet. In addition, significantly longer villus height, shorter crypt depth, and higher villus height to crypt depth ratio were observed in the jejunal and ileal tissues of chickens fed EP. EP supplementation upregulated the mRNA expression of NF-κB, TLR4, MyD88, IL-2, IFN-α, and IL-1β in the ileal and jejunal tissues (*P* < 0.05). Besides, we observed significantly higher (*P* < 0.05) short-chain volatile fatty acids (SCFAs) levels in the caecal contents of the EP group than in the control group. Furthermore, 16S-rRNA analysis revealed that EP supplementation altered gut microbiota and caused an abundance shift at the phylum and genus level in broiler chicken. Interestingly, we observed an association between microbiota and SCFAs production. Overall, this study demonstrated that supplementation of diet with EP promotes growth performance, improves intestinal immune response and integrity, and modulates the caecal microbiota of broiler chickens. This study highlighted the application of marine algae polysaccharides as an antibiotic alternative for chickens. Furthermore, it provides insight to develop marine algae polysaccharide-based functional food and therapeutic agent.

## Introduction

In the poultry industry, antibiotics have been used for long years as a feed additive to promote growth and control disease. However, recently, its use has been banned due to the risk of antibiotic-resistant microbes, residues in animal products, and environmental pollution (1). Therefore, there is an urgent need for antibiotic substitutes that can replace its functions while surmounting its shortcomings. In this regard, dietary fibers such as polysaccharides are known to improve health and homeostasis by enhancing the intestinal immune response and gastrointestinal barrier function (2). However, human and animal enzymes are unable to digest dietary fibers and are subject to fermentation by the gut microbiota (3). The gut microbiota lives in a symbiotic relationship with the host, in which the host provides habitat and nutrients for their growth, while the microbiota provides essential nutrients via the fermentation of fibers. Therefore, the dynamic diet-microbiota interactions shape the health and immune response of the host (4).

In chicken, caecum is the main organ that harbors a vast diversity of microbes responsible for the fermentation of fiber (5). These microbes possess polysaccharide-degrading enzyme-encoding genes and pathways involved in the production of short-chain volatile fatty acids (SCFAs), which are beneficial to the host physiology and energy homeostasis (6). The microbiota-derived SCFAs play an important role in maintaining an intestinal immune response, barrier function, and immune metabolism via the activation of metabolite-sensing G-protein coupled receptors (GPCRs) (7). Apart from metabolites production, gut microbiota plays a decisive role in maintaining the homeostasis and health of the host via direct involvement in gut structure and morphology, regulating immune responses, and protection from luminal pathogens (8). Therefore, changes in the type of polysaccharides consumed are expected to alter the composition and function of the microbiota, thereby altering the host immune system. Thus, understanding how a given microbial population responds to polysaccharides diet, and the role and association of this microbiota and its metabolites with immune response of the host play crucial roles to develop polysaccharide-based functional foods to prevent and treat gut microbiota-related diseases.

*Enteromorpha prolifera* (*E. prolifera*) is a seaweed green alga with a long history of use as food and traditional medicine. A sulfated polysaccharide is one of the main biologically active substances in *E. prolifera*, which is responsible for the immunomodulating, hypolipidemic, antitumor, anti-aging, antibacterial, anticoagulant, antiviral, and anticancer activities of these algae (9–12). *E. prolifera* polysaccharides (EP) are made up of α- and β-(1, 4)- linked monosaccharides (rhamnose, xylose, and glucuronic acid) (13), where the sulfate group is attached at the C-3 position of rhamnose (14). Recent studies indicated increased production performance, breast muscle yield, egg quality, antioxidant capacity, and intestinal morphology of chickens fed seaweed polysaccharides (15–18). In addition, EP supplementation increased the weight and differentially regulates the gene expression at the transcriptome level in the bursa of Fabricius of Arbor Acres chickens (19). Similarly, EP supplementation has been shown to improve the growth performance, non-specific immunity, and intestinal function of banana shrimp *F. merguiensis* fish (20). Furthermore, administration of EP was found to regulate intestinal microbiota in mice (21) and fecal microbiota in humans (22). Our recent study showed that supplementation of diet with EP- zinc complex could reduce diarrhea rate and improved intestinal barrier function in piglets (23). Although the biological activities of EP have been well-established so far, their interactions with gut microbiota in broiler chickens are largely unknown.

Considering the above information and the fact that polysaccharides are digested by the intestinal microbiota, we designed this study to investigate the effects of EP supplementation on growth performance, immune response, intestinal integrity, and gut microbiota in broiler chickens.

## Materials and Methods

### Source of *Enteromorpha* Polysaccharide

The EP was extracted from the marine algae *E. prolifera* and provided by Qingdao Seawin Biotechnology Group Co., Ltd. (Qingdao, China). The content of EP was not <45%, and the molecular weight was 4,431 Da. The water-soluble sulfated polysaccharides of EP were extracted from the *E. prolifera* by an enzymatic method according to the procedure previously described (13, 24). Briefly, the algae were washed with distilled water and dried at 60°C, then minced to get homogenate powder. The algal powders were soaked in water, and then the water extracts algae were subjected to stepwise enzymatic treatment with pectinase, cellulase, and papain at 50°C for 1.5 h. The enzyme reaction was inactivated by heating the reaction at 90–100°C for 10 min, and then immediately cooled on an ice bath, centrifugal concentrated, ethanol precipitation, and finally spray drying to obtain the polysaccharide products (25). The monosaccharide composition was determined using high-performance liquid chromatography (HPLC) according to the procedures previously described (14). Based on the HPLC analysis results, the monosaccharide composition of the EP used in this study was composed of rhamnose (Rha), glucuronic acid (GlcA), xylose (Xyl), glucose (Glc), and galactose (Gal) with the molar percentage of 40.6, 38.2, 9.3, 5.6, and 6.3%, respectively.

### Bird Management

The experimental design and procedures used in this study were reviewed and approved by the Animal Care and Use Committee of the Institute of Subtropical Agriculture, Chinese Academy of Sciences. The animal experiments and sample collection strictly followed the relevant guidelines. For this experiment, 200 healthy 1-day-old male Ross-308 broiler chickens were used. The chickens were kept in a room with 23-h of light and 1-h darkness. The room temperature was kept at about 32°C for 3 d and gradually reduced by 1°C every other day until the temperature reached 24°C, and then maintaining this temperature. The experimental chickens had access to *ad libitum* feed and water. All nutrients in experimental diets were formulated to meet or exceed the recommendations for Ross broiler chickens (26). The dietary composition and nutrient levels of the basal diet are presented in Table 1.

**Table 1:** Ingredient composition and nutrient contents of basal diets.

**Ingredients, %**	**1 ~ 21 d**	**22 ~ 42 d**
Corn	56.41	56.64
Soybean meal (CP, 43%)	29.85	28.30
Corn gluten meal	5.00	5.00
Soybean oil	3.90	5.30
Limestone	1.30	1.30
Dicalcium phosphate	1.80	1.75
_L_-Lysine	0.32	0.30
_DL_-Methionine	0.12	0.11
Premix^a^	1.00	1.00
Salt	0.30	0.30
Total	100	100
**Nutrient levels, %**		
Metabolizable energy, MJ/kg	12.86	13.20
Crude protein	21.12	19.40
Calcium	1.02	0.93
Available phosphorus	0.47	0.43
Lysine	1.20	1.06
Methionine	0.50	0.43
Methionine + cysteine	0.85	0.77
Arginine	1.35	1.33
Threonine	0.80	0.74

a *The premix provided per kilogram of diet: vitamin A, 15,600 IU; vitamin D3, 4,480 IU; vitamin E, 31 IU; vitamin B1, 2.4 mg; vitamin B2, 7.2 mg; vitamin B6, 6.3 mg; vitamin B12, 0.32 mg; niacin, 47 mg; pantothenic acid, 16.2 mg; folic acid, 1.6 mg; biotin, 0.26 mg; Cu, 10.4 mg; Zn, 83.2 Fe, 75 mg; Mn, 83.1 mg; Se, 0.5 mg; I, 0.5 mg*.

### Diet and Experimental Design

For this experiment, 200-1-day-old male broiler chickens were randomly divided into two treatment groups with ten replications of ten chickens per replication. The first group was fed a basal diet (control group) and the treatment group received a basal diet supplemented with 400 mg EP/kg diet (EP group), according to the dose recommended (15). The experiment lasted for 42 days.

### Sample Collection

The feed offers, leftover, and body weight were recorded to calculate the average daily feed intake (ADFI) and average daily gain (ADG). Feed conversion ratio (FCR) was calculated as ADFI/ADG. At the end of the experiment (day 42), blood samples were collected from one chicken from each replication (*n* = 10/treatment). The sera were separated by centrifuging at 3,000 rpm for 15 min at 4°C and stored at −20°C for subsequent analysis. Thereafter, one chicken from each replication (*n* = 10/treatment) close to the average body weight of the group was humanely euthanized by cervical dislocation for tissue samples collection.

Small intestinal tissues (jejunum and ileum) were isolated. The middle sections of small intestinal tissues were then fixed in 4% formaldehyde for morphological analysis. The other half of the small intestinal tissues were immediately frozen in liquid nitrogen and stored at −80°C for gene expression analysis. Caecal contents were collected and frozen for microbiota and volatile fatty acids analysis.

### Serum Cytokine Analysis

Serum concentrations of interleukin-1β (IL-1β), IL-2, IL-6, IL-10, tumor necrosis alpha (TNF-α), and interferon-gamma (INF-γ) were measured using commercial chicken-specific ELISA kits (Shanghai Kexin Biotech Co., Ltd, Shanghai, China), following the kit instruction.

### Morphological Analysis of Small Intestinal Tissues

The paraformaldehyde-fixed segment of the jejunum and ileum tissues were embedded in paraffin, sectioned (5 μm), and stained with hematoxylin and eosin as previously described (27). Villus height was then measured from the tip of the villus to the top of the lamina propria, and crypt depth was measured from the villus-crypt axis to the tip of the muscular mucosa. The villus height to crypt depth ratio was then calculated.

### mRNA Expression Analysis of Immune-Related Genes and Tight Junction Molecules

Quantitative real-time polymerase chain reaction (RT-qPCR) was used to investigate the effects of EP supplementation on immunity and intestinal integrity-related genes expression. Briefly, total RNA was isolated from the frozen intestinal tissues using a trizol reagent (Invitrogen Co., CA, USA) and then treated with DNase I (Invitrogen, Carlsbad, CA, USA) according to the manufacturer instructions. The integrity was detected by 1% agarose gel electrophoresis, and the quality and quantity were assessed using Nanodrop 2000 (Thermo Fisher Scientific, Waltham, MA, USA). The cDNA was then synthesized using EvoM-MLV RT kit (Accurate Biotechnology, Hunan, China) according to the kit instructions. The RT-qPCR was performed on Roche LightCycler^®^ 480II (Roche, Basel, Switzerland) using SYBR Green mix (Takara, Tokyo, Japan) with targets and β-actin (housekeeping) genes primers (Table 2). Thermal cycling conditions were initial denaturation of 95°C for 30 s, followed by 40 amplification cycles of 95°C for 15 s, 60°C for 30 s, and 72°C for 60 s. The gene expression levels were recorded as the threshold cycle (CT) values that corresponded to the number of cycles at which fluorescence signals can be detected. The relative mRNA expression of genes was calculated using the 2^−ΔΔCt^ methods described previously (28).

**Table 2:** Primers used for quantitative polymerase chain reaction.

**Gene name**	**Accession no**	**Primer 5′-3′**
*IL-2*	AF000631	F: TCTGGGACCACTGTATGCTCT
		R: ACACCAGTGGGAAACAGTATCA
*IL-10*	AJ621614	F: CGGGAGCTGAGGGTGAA
		R: GTGAAGAAGCGGTGACAGC
*IL-1β*	Y15006	F: GTGAGGCTCAACATTGCGCTGTA
		R: TGTCCAGGCGGTAGAAGATGAAG
*TNF-α*	AY765397	F: TGCTGTTCTATGACCGCC
		R: CTTTCAGAGCATCAACGCA
*IFN-γ*	NM_205149.1	F: TGAGCCAGATTGTTTCGA
		R: ACGCCATCAGGAAGGTTG
*TLR-2*	AB046119.2	F: GGGGCTCACAGGCAAAATC
		R: AGCAGGGTTCTCAGGTTCACA
*TLR4*	AY064697	F: AGTCTGAAATTGCTGAGCTCAAAT
		R: GCGACGTTAAGCCATGGAAG
*MyD88*	EF011109	F: TGATGCCTTCATCTGCTACTG
		R: TCCCTCCGACACCTTCTTTCTA
*NF-kB*	NM_205129	F: TCCCTCCCGACGAATTTTGG
		R: CTGACACTGCACCAAACGTG
*Occludin 1*	NM_205128.1	F: ACGGCAGCACCTACCTCAA
		R: GGGCGAAGAAGCAGATGAG
*Claudin 1*	NM_001013611.2	F: CATACTCCTGGGTCTGGTTGGT
		R: GACAGCCATCCGCATCTTCT
*ZO2*	NM_204918.1	F: GGATACAATTCAGCAACAGCAAGG
		R: ACATGCGATCATCTGCGTCATCT
*Mucin 2*	XM_421035	F: AGGTAATTGTCTGGCCGTGG
		R: GTGGTTGTACCTTCGGTGCT
*β-actin*	NM 205518.1	F: ACCGGACTGTTACCAACACC
		R: CCTGAGTCAAGCGCCAAAAG

### Microbiota Profiling

Microbial DNA was isolated from the caecal content of six chickens in each group using the E.Z.N.A. Stool DNA Kit (D4015, Omega, Norcross GA, USA) according to the manufacturer's instructions. Following the extraction, the quality and quantity of DNA were assessed using NanoDrop 2000 spectrophotometer (Thermo Scientific, Waltham, MA, USA) and integrity was checked using 1% agarose gel electrophoresis. Then, the V3–V4 region of the bacterial 16S-rRNA gene was amplified by PCR using the primers (F: 5′-ACTCCTACGGGAGGCAGCAG-3′; R: 5′-GGACTACHVGGGTWTCTAAT-3′). The PCR product was run on 2% agarose gel, and then excised and purified using the AxyPrep DNA Gel Extraction Kit (Axygen Biosciences, Union City, CA, USA) and quantified using Quantus™ Fluorometer (Promega, Madison, USA). DNA libraries were constructed using the TruSeq^®^ DNA PCR-Free Sample Preparation Kit (Illumina, San Diego, USA). The sequencing library was evaluated on the Qubit^®^ 2.0 Fluorometer and Agilent Bioanalyzer system (Thermo Fisher Scientific, Waltham, MA, USA) and then subjected to paired-end sequencing on an Illumina HiSeq platform at Novogene Bioinformatics Technology Co., Ltd. (Beijing, China). Paired-end reads from the original DNA fragments were merged by FLASH and resulting labels were assigned to the Operational Taxonomic Units (OTUs) with a threshold value of 97% using UPARSE (http://drive5.com/uparse). The species diversity (α-diversity) Chao, Shannon, and Simpson indices were estimated using QIIME2. Linear discriminant analysis (LDA) effect size (LEfSe) was performed to reveal the difference in the bacterial communities across the treatments using the non-parametric factorial Kruskal-Wallis test with an alpha value of 0.05 and LDA score of 2.5. In addition, the relative abundance of dominant bacteria at the phylum and genus levels was also analyzed. Spearman correlation was used to investigate the association between gut microbiota and SCFAs production.

### Short-Chain Fatty Acids Analysis

The short-chain volatile fatty acids (acetate, butyrate, propionate, iso-butyrate, valerate, and iso-valerate) were determined from caecal digesta samples using the Agilent 6,890 gas chromatography (Agilent Technologies, Inc, Palo Alto, CA) according to the previous study (29).

### Data Analysis

All data except the 16S-rRNA were analyzed using the statically analytical software (SAS 9.1 Institute, Inc., Cary, NC, USA). The growth performances, cytokines, mRNA expression, and caecal SCFAs content data were checked for normality and homoscedasticity of the data variance using the Shapiro-Wilk test and Levene's test, respectively, and then subjected to an independent *t*-test. The data are presented as the mean ± standard error of the mean (SEM) and statistically significant was considered when *P* < 0.05.

## Results

### Growth Performance

The effects of EP supplementation on the growth performances of chickens are shown in Table 3. Results showed that chickens fed a diet supplemented with EP exhibited significantly higher (*P* < 0.05) body weight and average daily gain compared with the control group. There were no significant differences in feed intake (FI) and feed conversion ratio (FCR) between the treatment groups (*P* > 0.05).

**Table 3:** The effects of EP supplementation on growth performance of broiler chickens.

**Parameters**	**Treatment groups**		***P-Value***
	**Control**	**EP**	
Initial BW, g	41.14 ± 0.25	41.03 ± 0.22	0.94
Final BW, g	2,145.14 ± 32.01^b^	2,243.11 ± 30.27^a^	0.038
ADG, g/day	50.10 ± 0.76^b^	52.43 ± 0.72^a^	0.039
FI, g/day	86.61 ± 1.94	87.40 ± 0.63	0.57
FCR	1.73 ± 0.02	1.67 ± 0.03	0.39

*BW, body weight; ADG, average daily gain; FI, feed intake; FCR, feed conversion ratio*.

*Data are presented mean ± SEM; n = 10. Means across a row with different superscript letter denotes significant different at P < 0.05*.

### Dietary EP Supplementation Regulates Serum Cytokine Levels

**Figure 1:**
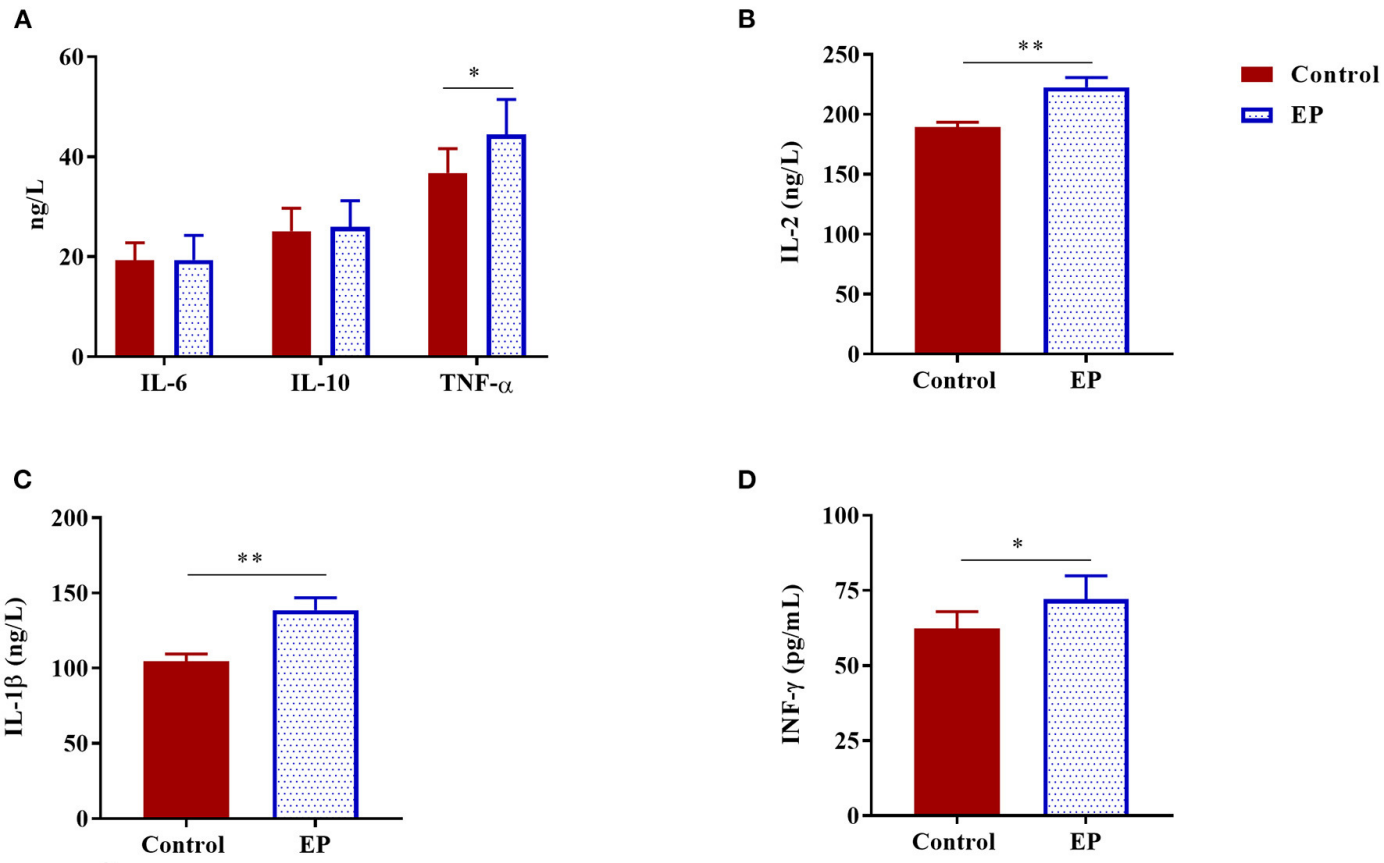
The effects of dietary EP supplementation on serum cytokine levels of broiler chickens. Data are presented as mean ± SEM, *n* = 10. *P < 0.05 and **P < 0.01, respectively. **(A)** IL-6, Interleukin 6; IL-10, Interleukin 10; TNF-α, Tumor necrosis factor-alpha; **(B)** IL-2, Interleukin 2; **(C)** IL-1β, Interleukin 1 beta; **(D)** IFN-γ, Interferon-gamma.

To better understand the effects of EP supplementation on immune response, we measured six common cytokines in sera obtained from experimental chickens (Figure 1). Compared with the control chickens, a significant increase in serum levels of IL-1β, IL-2, TNF-α, and IFN-γ (*P* < 0.05) were observed in chickens fed the basal diet supplemented with EP. In this study, EP supplementation did not affect the serum IL-6 and IL-10 levels in broiler chickens.

### Intestinal Morphology Analysis

**Figure 2:**
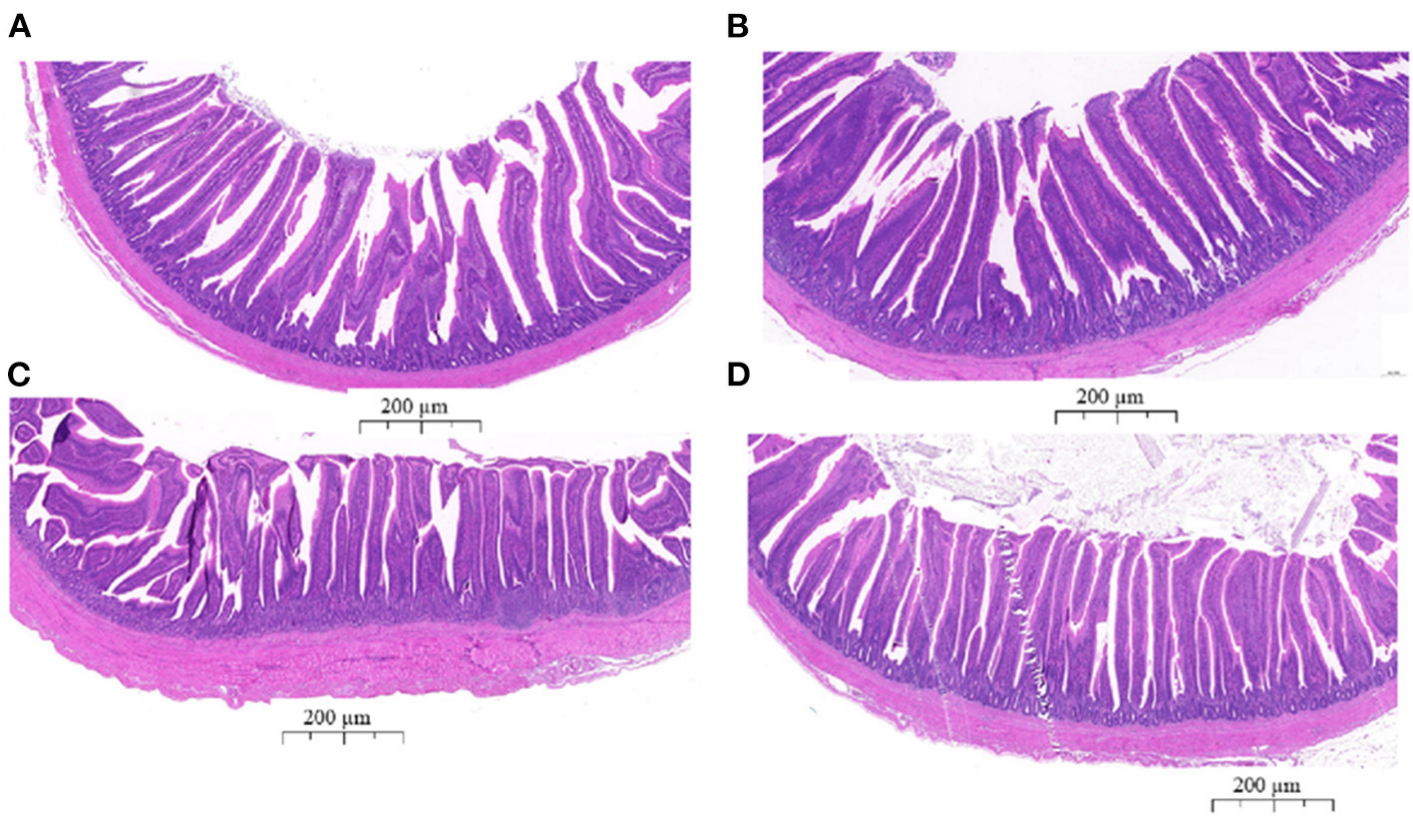
The histological analyses of the jejunum and ileum tissues from the control group **(A,C)** and EP supplemented **(B,D)** broiler chickens stained with hematoxylin and eosin. **(A)** Jejunum tissue from the control group; **(B)** jejunum tissue from the EP group; **(C)** Ileum tissue from the control group; **(D)** Ileum tissue from the EP group. The scale bar represents 200 μm.

To determine the effects of EP supplementation on intestinal morphology, jejunal and ileal tissues were fixed using hemotoxin and eosin (Figure 2). In the jejunum, longer villus height and shorter crypt depth ratio were observed in the EP supplemented group (*P* < 0.05; Table 4). Similarly in the ileum, villus height and villus height: crypt depth ratio were significantly (*P* < 0.05) increased, while crept depth was markedly reduced (*P* < 0.05) in the EP supplemented group than in the control group (Table 4). However, a significant difference in the villus height: crypt depth ratio between treatment groups was not observed (*P* = 0.064) in the jejunal tissue.

**Table 4:** Effects of EP polysaccharide supplementation on the intestinal morphology of broiler chickens.

**Parameters**	**Treatment groups**		***P-*Value**
	**Control**	**EP**	
**Villus height (μm)**			
Jejunum	1,094.61 ± 29.13^b^	1,207.51 ± 65.47^a^	0.017
Ileum	855.93 ± 45.98^b^	1,038.91 ± 46.96^a^	0.008
**Crypt depth(μm)**			
Jejunum	238.08 ± 7.83^a^	201.23 ± 7.51^b^	0.03
Ileum	210.43 ± 16.83^a^	172.28 ± 12.13^b^	0.013
**VH/CD**			
Jejunum	4.6 ± 0.20	6.0 ± 0.44	0.063
Ileum	4.11 ± 0.24^b^	6.20 ± 0.43^a^	0.020

*VH/CD, villus height: crypt depth ratio; n = 10 for each group. Means with a different superscript letter in the same raw indicate a significant difference at P < 0.05*.

### Effects of EP Supplementation on Immune-Related Gene Expression

**Figure 3:**
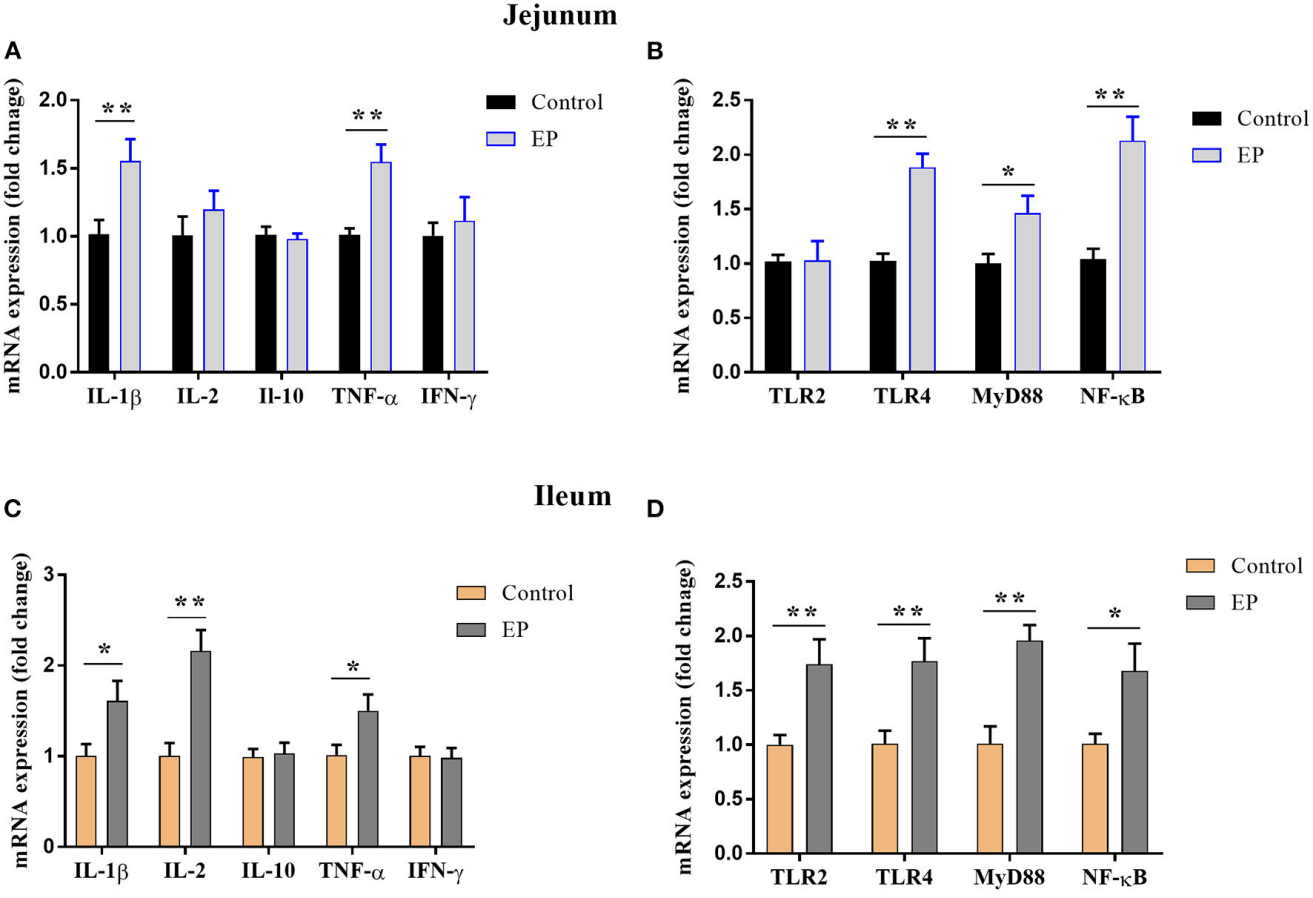
Effects of EP supplementation on the expression of immune factor-related genes **(A,C)** and inflammatory signaling pathway genes **(B,D)** in the jejunum tissues **(A,B)** and ileum **(C,D)** of broiler chickens. Data are presented as mean ± SEM, *n* = 10. *P < 0.05 and **P < 0.01, respectively. IL-1β, Interleukin 1 beta; IL-2, Interleukin 2; IL-10, Interleukin 10; TNF-α, Tumor necrosis factor-alpha; IFN-γ, Interferon-gamma; TLR, Toll-like receptor; MyD88, myeloid differentiation 88; NF-κB, Nuclear factor-kappa B.

To determine the effects of EP supplementation on intestinal immune response, we detected the mRNA expression of immune-related genes from the jejunal and ileal tissues. In the jejunum, compared with the control group, EP supplementation upregulated (*P* < 0.05) the mRNA expression of IL-1β, TNF-α, TLR4, MyD88, and NF-κB (Figures 3A,B). However, dietary EP inclusion did not significantly alter the mRNA expression of IL-2, IFN-γ, IL-10, and TLR2 in the jejunal tissue (Figures 3A,B; *P* > 0.05).

In the ileum, the mRNA expressions of IL-1β, IL2, and TNFα were upregulated (*P* < 0.05) in the EP group than in the control group (Figure 3C). In addition, the expressions of Toll-like receptor (TLR-2), TLR4, myeloid differentiation (MyD88), and nuclear factor-kappa B (NF-κB) in the ileum tissue were significantly increased (*P* < 0.05) in the supplemented group (Figure 3D). However, EP supplementation did not affect (*P* > 0.05) the mRNA expression of IFN-γ and IL-10.

### Effects of EP Supplementation on Mucin-2 and Tight Junctions Gene Expression

**Figure 4:**
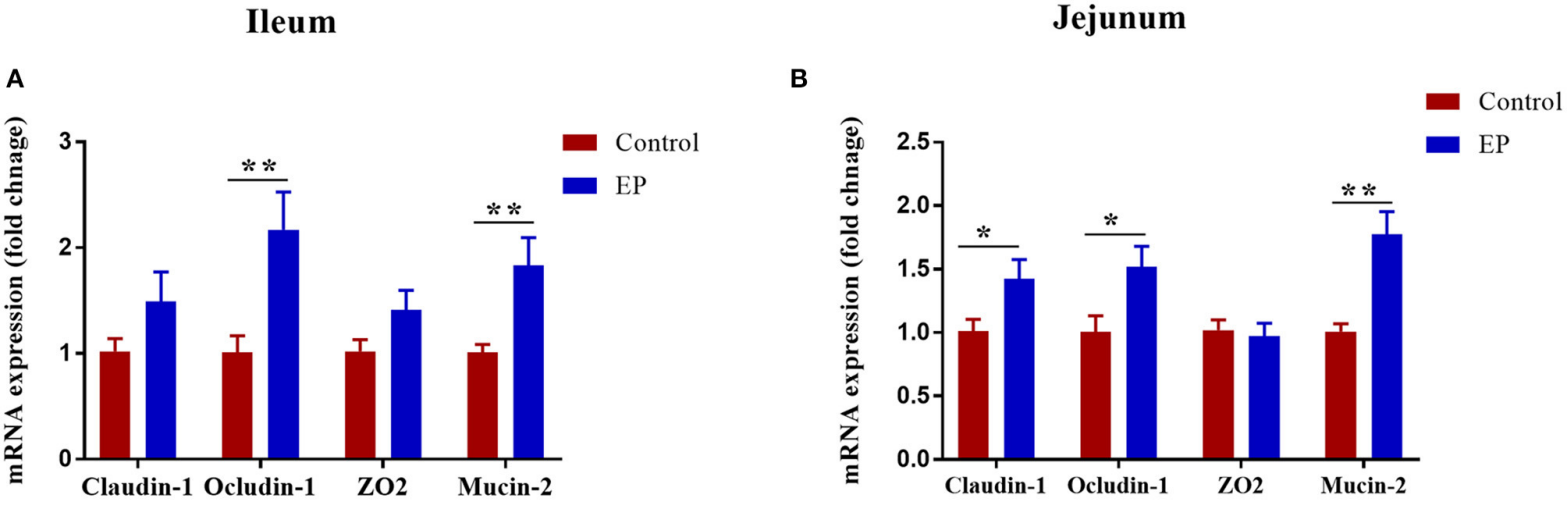
Effects of EP supplementation on Mucin-2 and tight junction gene expression in the ileum **(A)** and jejunum **(B)** tissues of broiler chickens. Data are presented as mean ± SEM, *n* = 10. *P < 0.05 and **P < 0.01, respectively.

The effects of EP supplementation on mRNA expression of mucin-2 and tight junction in the jejunum and ileum tissues are presented in Figure 4. In the jejunum, the addition of EP to diet upregulated (*P* < 0.05) the mRNA expression of mucin-2, claudin-1, and occludin-1. In the ileum, birds fed EP had a significantly higher expression of mucin-2 and occludin-1 (*P* < 0.05) than the control group. However, no differences (*P* > 0.05) were observed between the treatments in ZO2 expression in the jejunum, and claudin-1 and ZO2 in the ileum.

### Effects of EP Supplementation on Microbiota Dynamics

**Figure 5:**
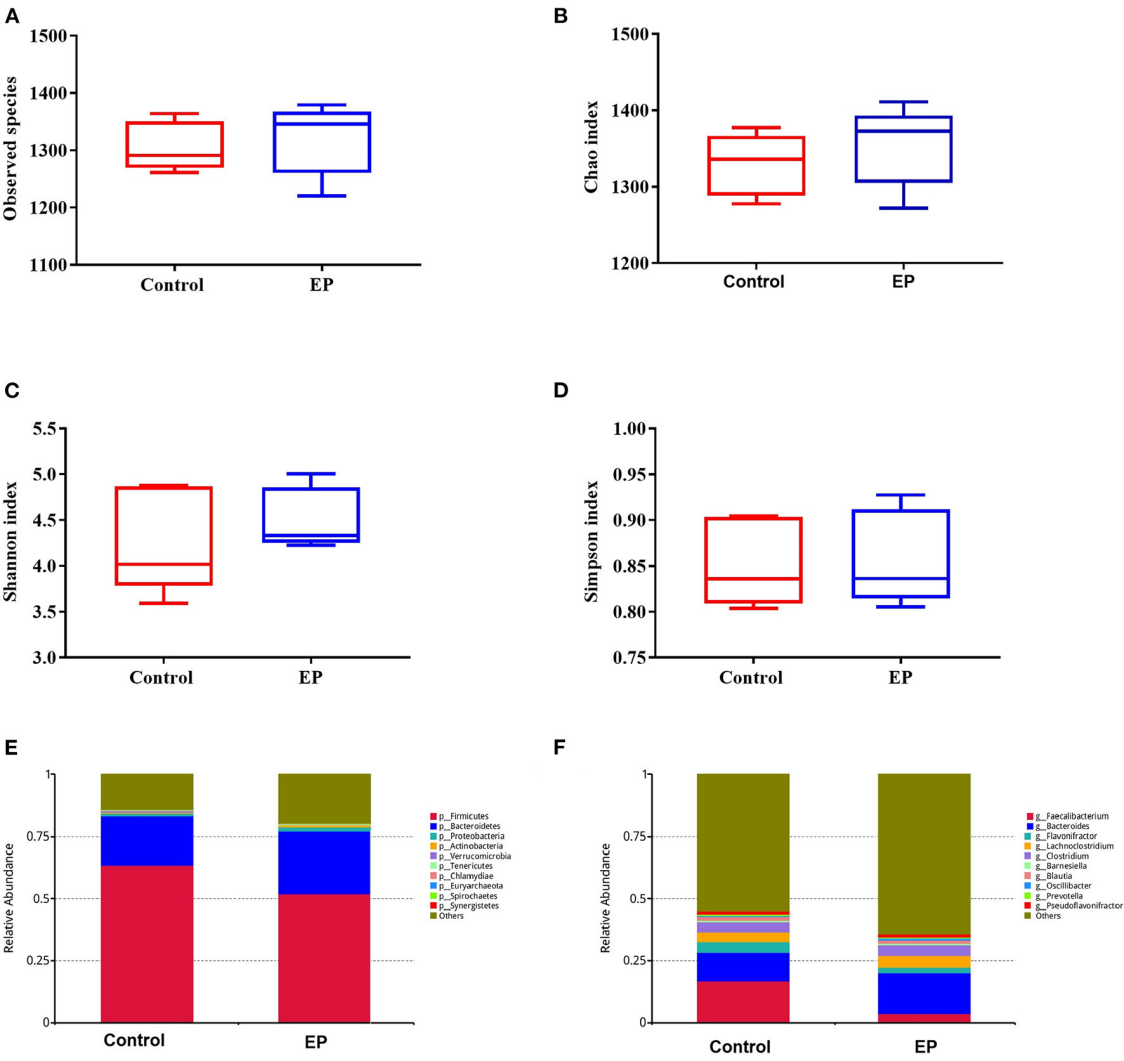
The alpha diversity and the relative abundances of caecal microbiota from the EP and control groups broiler chickens. The number of observed species **(A)**, Chao index **(B)**; Shannon index **(C)**; Simpson index **(D)**; the relative abundance of major bacterial phyla **(E)**; and major bacterial genus **(F)** between the EP and control groups. EP, *Enteromorpha prolifera* polysaccharide.

To assess the caecal microbial composition in response to EP supplementation, the caecal contents of 10 experimental broiler chickens were collected and subjected to metagenomic sequencing. We retrieved 60,537.45 and 6,053.75 Mbp total raw and average raw reads, respectively. After quality control, we obtained 58,156.5 and 5,815.65 Mbp total and average clean data, respectively. To explore the differences in species diversity and richness between EP supplemented and control groups, we calculated the alpha diversity indexes at the phylum level. The results showed that there were no significant differences in observed species, Chao, Shannon, and Simpson indexes between treatment groups [Mann–Whitney *U* (MWU) (Figures 5A–D; *P* > 0.05].

**Figure 6:**
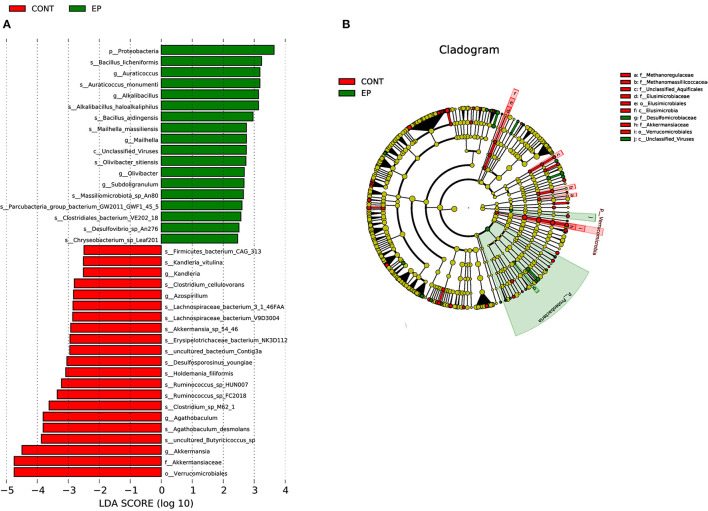
Linear discriminant analysis (LDA) effect size (LEFSe) analysis identified microbial taxa between EP (green) and (control (red) groups. **(A)** histogram plot from LEfSe analysis that presents the LDA scores of microbial taxa whose abundance showed significant differences between EP supplemented and control group broiler chickens (LDA score > 2.5). The length of the bar column represents the LDA score. **(B)** The cladogram, circles radiating from inner side to outer side represents the differences in the relative abundance of taxa from phylum to genus level between EP and control group. The red and green dots indicate a significant difference in the relative abundance between EP and the control group.

The gut microbiota is composed of different bacterial species and is classified according to genus, family, order, and phyla. Therefore, analyzing their composition helps to identify specific microorganisms involved in different processes and the associated metabolic pathways. Thus, we analyzed the microbial abundance from taxonomic phylum to genus levels in different groups. We found that *Firmicutes* and *Bacteroidetes* are the two most predominant phyla, which accounted for more than 75% of the microbes observed (Figure 5E). The results further demonstrated that EP supplementation reduced the relative abundance of *Firmicutes* and decreased *Bacteroidetes* microbes. Furthermore, an increase in the relative abundance of *Bacteroides* and decreased *Faecalibacterium* were observed at the genus level (Figure 5F). The difference in bacterial abundance between treatment groups was estimated using linear discriminant analysis (LDA), which could be used as a biomarker. In total, 38 phylotypes from phylum to species were identified as high-dimensional biomarkers with LDA scores >2.5 (Figure 6) Remarkably, the species *Bacillus_licheniformis, Auraticoccus_monumenti*, and *Alkalibacillus_haloalkaliphilus* were biomarkers in the EP group, while *uncultured_Butyricicoccus_sp, Agathobaculum_desmolans*, and *Clostridium_sp_M62_1* were predominant in the control group.

### Effects of EP Supplementation on the SCFAs and Correlation Analyses Between SCFAs and Gut Microbiota

**Figure 7:**
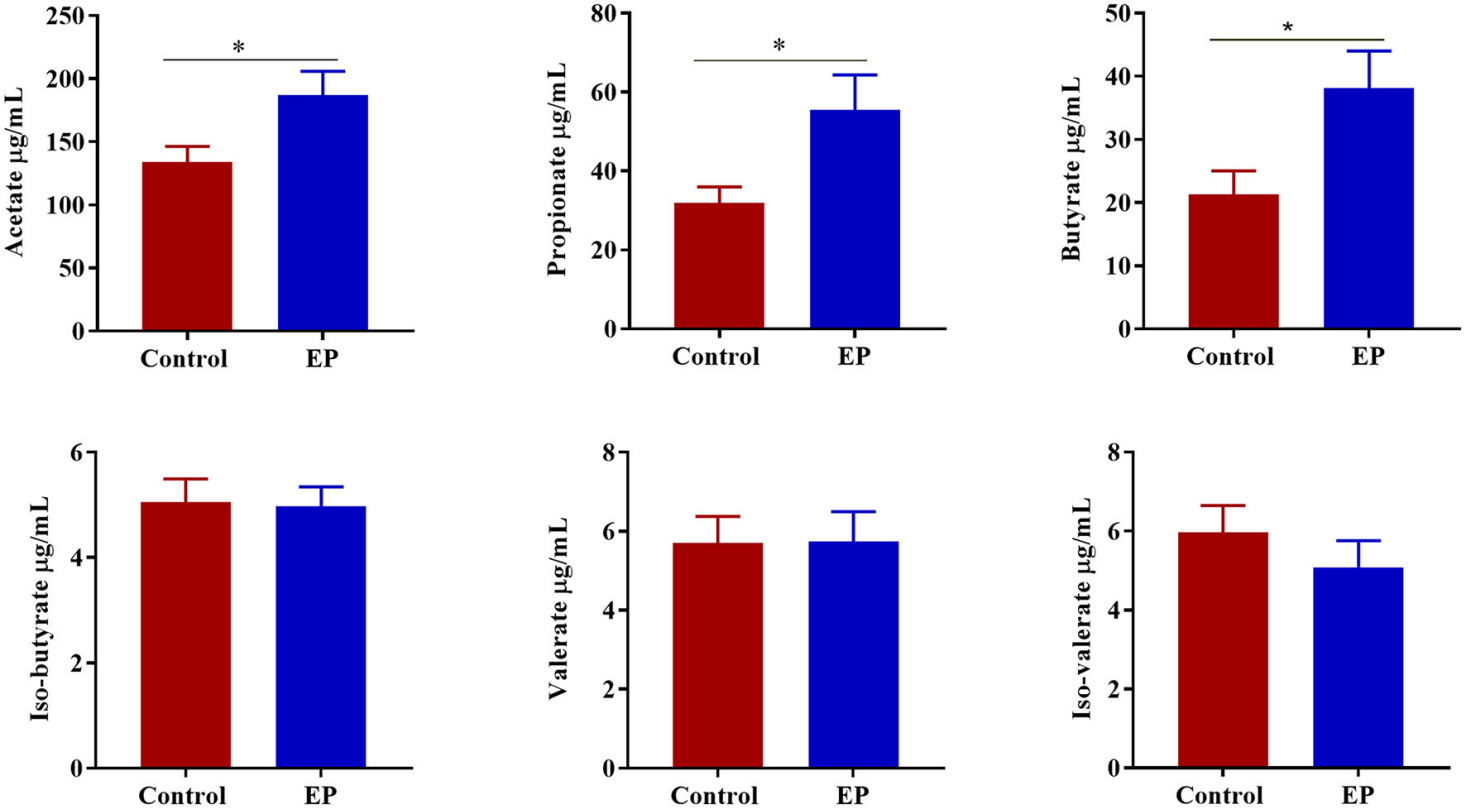
The effects of EP supplementation on the short chain volatile fatty acid concentration from caecal content of broiler chickens. *P < 0.05.

To determine the effects of EP supplementation on SCFAs production, we measured the concentrations of SCFAs from caecal content (Figure 7). Compared with the control group, chickens fed a diet supplemented with EP had significantly higher (*P* < 0.05) acetate, butyrate, and propionate levels in the caecal content. However, a significant treatment effect (*P* > 0.05) on the valerate, iso-butyrate, and iso-valerate content was not observed.

**Figure 8:**
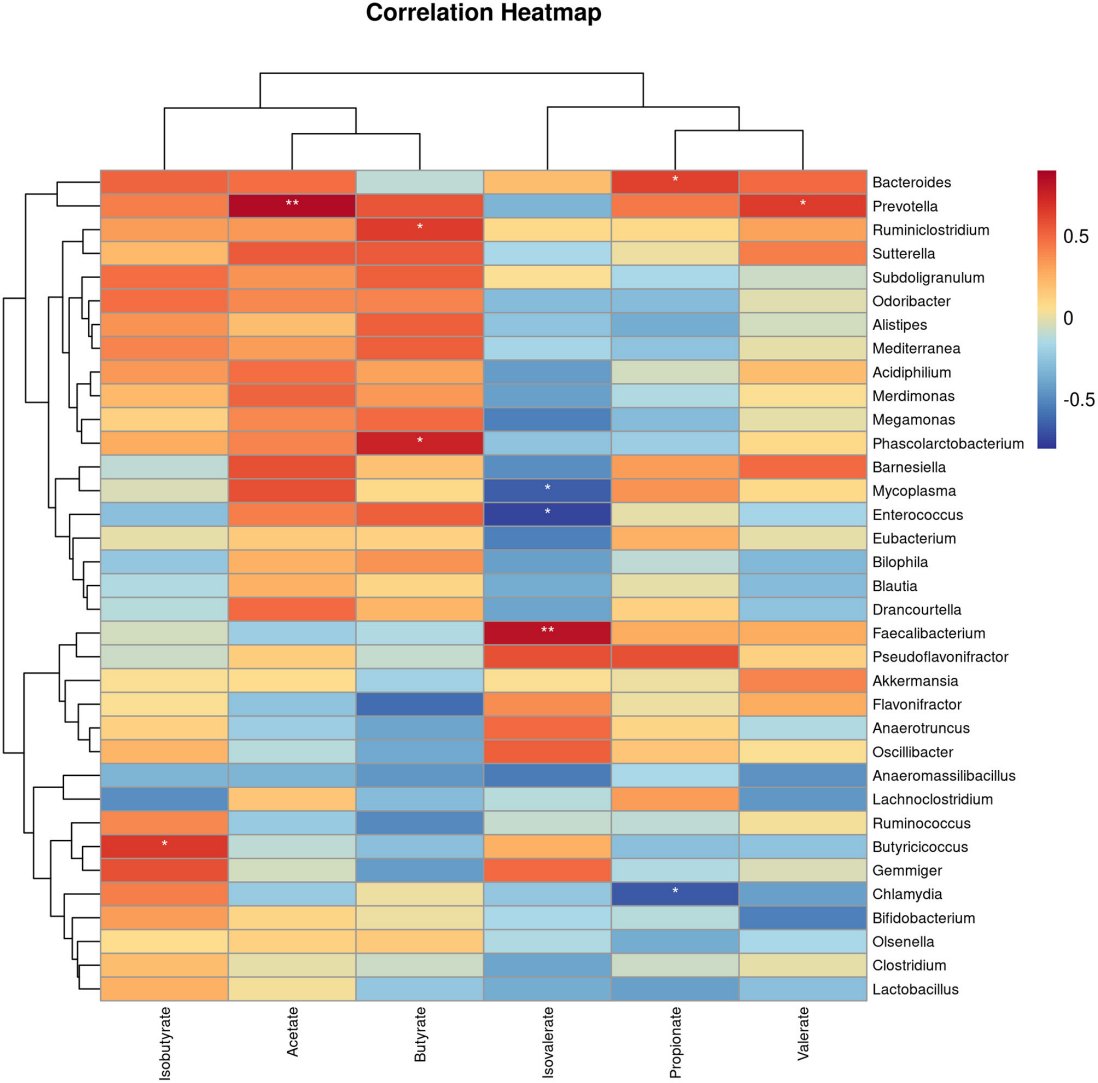
The heat map represents the correlation between caecal SCFAs and caecal microbiota in EP and control group broiler chickens. *P < 0.05 and **P < 0.01, respectively.

To gain insight into whether the altered gut microbiota had an association with the SCFAs, we carried out Spearman correlation analyses. The relationships of the altered gut microbiota and cecal SCFAs in response to EP treatment based on the Spearman correlation coefficients are shown in Figure 8. Notably, the relative abundance of the *Bacteroides, Prevotella, Ruminiclostridium, Butyricicoccus*, and *Faecalibacterium* had a significant positive association with propionate, acetate, butyrate, isobutyrate, and isovalerate production, respectively (*P* < 0.05). In addition, *Prevotella* had also a positive association with valerate (*P* < 0.05). In contrast, the abundance of *Chlamydia* had a negative association with propionate, and *Enterococcus* and *Mycoplasma* had a negative association with isovalerate (*P* < 0.05).

## Discussion

Apart from its use as a food and traditional medicine, recent studies have shown that sulfated polysaccharides from marine algae exerted various biological activities, such as immunomodulation, antioxidant, antidiabetic, and hypolipidemic. In the present study, we supplemented the broiler diet with polysaccharides isolated from marine algae EP to evaluate its effect on growth performance, immune response, intestinal barrier function, and caecal microbiota.

Herein, chickens fed EP exhibited higher body weight and average daily gain than chickens fed only basal diet, suggesting that EP supplementation to broiler diet could improve growth performance. Similar conclusions were made by Liu et al. (16) and Li et al. (30), reported that algae-derived polysaccharide could enhance growth performance in chicken. Likewise, dietary supplementation of EP was found to improve growth performance in mice (31) and crucian carp (32). The growth-promoting effect of EP may be associated with its enhancement of nutrient absorption by improving intestinal function and morphology (31).

Research evidence has shown that polysaccharide from *E. prolifera* has an immunomodulatory activity (33). Cytokine profiling is a valuable tool for monitoring immune responses associated with inflammation and immunity. In this study, supplementation of diet with EP induced a profound change in the serum cytokine contents of broilers as evidenced by significantly higher IL-1β, IL-2, IFNα-, and IFN-γ levels in the supplemented group. This study is consistent with the previous study that reported EP could stimulate proinflammatory cytokine production in mice (34). The results of the present study also support the idea that EP has an immunomodulatory activity by stimulating proinflammatory cytokines.

To better understand whether the changes in cytokine levels were accompanied by changes in the expression of inflammatory factor genes, we detected mRNA expression in the jejunum and ileum tissues. We found upregulated mRNA expression levels of IL-1β and TNF-α in the ileum and jejunum, and IL-2 in the ileum in the EP group compared with the control group. In contrast, Liu et al. (35) reported that algae-derived polysaccharides down-regulated the mRNA expression of TNF-α, and IL-6 in the bursa of Fabricius of heat stress chicken. The discrepancy of our study with the previous study could attribute to the difference in immune status and stress condition of chicken.

More importantly, the pro-inflammatory pathway is transcriptionally regulated by NF-κB, and the up-regulation of NF-κB activates the immune response and cytokine production. The TLR4/MyD88 signaling pathway is the upstream gatekeeper of NF-κB (36). The present data showed that the expression of TLR4, MyD88, and NF-κB in ileum and jejunum were significantly higher in chicken-fed EP than in chicken-fed only basal diet. TLR4 is a family of pathogen recognition receptors (PRRs) that orchestrate the host immune system through MyD88 to induce pro-inflammatory cytokines via NF-κB (37, 38). As the downstream of the TLR4 and interleukin-1 (IL-1) receptor, MyD88 activates NF-κB, and thereby the inflammatory signaling pathways (39). Therefore, the present study indicated that EP supplementation activates the TLR4/MyD88/NF-κB signaling pathway, thereby induces an immune response. This observation further supported the viewpoint that EP has immunomodulatory activity. Similarly, Wei et al. (33) reported upregulation of NF-κB transcription factors in mice that received EP. In contrast, a study on oxygen-glucose deprivation-induced human cardiac microvascular endothelial cells showed that EP inhibits proinflammatory factors expression through the up-regulation of HIF-1α and inactivation of the NF-κB pathway (40). This may be due to EP might play both immunomodulatory and anti-inflammatory roles depending on the physiological condition of experimental animals or cells.

Intestinal morphology affects nutrient absorption in the body. The longer intestinal villi and lower crypt depth are an indicator of higher nutrient absorption (41). The present study demonstrated that EP supplementation increased villus height and reduced crypt depth in the ileum and jejunum of supplemented chicken. The longer villus height and higher villus height: crypt depth ratio is associated with active cell mitosis, improved nutrient digestibility, and absorption in chickens (42, 43). Thus, the present study suggests the beneficial effects of EP supplementation on improving intestinal morphology. These results are in agreement with the study by Liu et al. (16), who reported villus height and villus height: crypt depth was higher in chickens fed algae-derived polysaccharides. Similar results were obtained by Guo et al. (15), who showed that marine-derived polysaccharide enhanced jejunal villus height and villus height/crypt depth ratio in layer chickens.

The mucus layer of the epithelial cells is an essential first line of defense that forms a barrier to prevent the penetration of the epithelium by gut microorganisms (44, 45). In addition, epithelial barrier integrity is maintained by the tight junction proteins, including occludin, claudins, and zonula occludens. Herein, we examined the effects of EP supplementation on intestinal barrier function by measuring the mRNA expression of mucin-2 and tight junction proteins. Our data demonstrated that the mRNA expressions of mucin-2 and occludin 1 in the ileum and jejunum were upregulated in the EP supplemented chickens. mucin-2 plays an important role in the secretion of mucus, which is a layer that protects the epithelial cells from exposure to the microbiome (46). Occludin provides structural integrity and assembly of tight junction and knockdown of occludin induces an increase in paracellular permeability to macromolecules (47). Claudins are also playing an essential role in barrier formation and paracellular permeable selectivity (48). Therefore, an increase in the expression of mucin-2, occludin 1, and claudin 1 in the intestinal segments in this study, indicates EP supplementation improved intestinal integrity in broiler chickens via regulating mucin-2 and tight junction protein. This is partly explained by the fact that *Enteromorpha* polysaccharides may be directly recognized by the pattern recognition receptors of intestinal epithelial cells (31, 49), leading to the activation of mucin-2 and tight junction proteins, thereby improving intestinal integrity. This idea is reinforced by the mRNA expression results of TLR4 and TLR2 in jejunum and ileum. In addition, EP was fermented by caecal microtia and produced SCFA, which could stimulate intestinal goblet cells to secrete mucin-2 through their action on NOD-like receptor family pyrin domain containing 6 (NLRP6) and G protein-coupled receptors (GPCRs) (50).

In broiler chickens, TLR2 is the principal receptor for peptidoglycan from gram-negative and gram-positive bacteria (51). Toll-like receptor 2 signaling has been implicated in preserving intestinal barrier integrity and is considered to be a crucial target for therapeutic intervention of metabolic and inflammatory conditions (52). In the present study, we found a significant increment in the mRNA expression of TLR2 in the ileum tissue of chicken fed EP. This result indicates that apart from the mucin-2 and tight junction protein, TLR2 might also be involved in the improvement of intestinal integrity observed in this study.

The fermentation of fiber diet by gut microbiota produced metabolites such as short-chain volatile fatty acids. These microbiota-derived short-chain fatty acids play important roles in maintaining an intestinal immune response, barrier function, and immune metabolism either by modulating gene transcription or via the activation of metabolite sensing GPCRs (53). In the current study, we found that the SCFAs particularly acetate, butyrate, and propionate increased in the caecal content of EP supplemented chickens, suggesting that EP enhanced the ability of the microbiota to induce SCFAs. Furthermore, we found significant correlations of differentially abundant gut microbes with acetate, propionate, and butyrate production, which further confirmed that the increase of SCFA observed in this study was due to the microbiota compositional shift. In agreement with the current study, mice fed EP showed an increase in SCFAs production in the colon (21). The SCFAs, particularly butyrate and propionate, provide energy for the immune cell by activating intestinal gluconeogenesis, thereby improving inflammatory and effector cytokines production and antigen presentation (54, 55). Furthermore, microbial-derived butyrate was found to promote mucosal barrier integrity by stimulating the production of mucin-2, tight junction proteins, and antimicrobial peptides (56). Therefore, the immune-modulatory role of EP in the current study might be via enhancing the gut microbiota-derived SCFA that contribute to intestinal immune response and gut barrier function.

A growing body of evidence suggests that gut microbiota is involved in the digestion and utilization of fibers such as polysaccharides, which otherwise cannot be utilized by the host. It is well-established that diet and nutritional factors have a direct effect on the microbial colonization of the gut (57–59). In this study, we conducted 16S-RNA sequencing from the caecal content of chickens fed EP to investigate microbiota dysbiosis. We found that the abundances of phylum *Bacteroidete*s and genera *Bacteroides* were increased, whereas phylum *Firmicutes* and genus *Faecalibacterium* were decreased in chickens fed EP, suggesting that *Bacteroides* might involve in the digestion of *E. prolifera* polysaccharides. This may be explained by the fact that *Bacteroides* possess genes encoding carbohydrate-active enzymes (CAZymes) within their polysaccharide utilization loci (PUL) (60, 61), which could confer them with the strong ability to ferment diverse types of dietary polysaccharides (62). Apart from polysaccharide fermentation, *Bacteroides* can also affect host immune system development (63), and maintenance of gut microbial balance (64). The present study is concurrent with the previous study that showed *E. prolifera* polysaccharides supplementation caused microbiota dysbiosis in mice (21) and rabbitfish *S. oramin* (65). These findings suggest that EP supplementation shifts microbiota composition, particularly *Bacteroides*, which produce SCFA that involve in immune response and gut barrier function. Notably, the species *Bacillus_licheniformis, Auraticoccus_monumenti*, and *Alkalibacillus_haloalkaliphilus* were biomarkers in the EP group. Previous studies reported that *B. licheniformis* isolated from a human fecal sample can be used for manufacturing biochemicals, enzymes, antibiotics, and aminopeptidase (66, 67). *Alkalibacillus_haloalkaliphilus* is used in enzyme synthesis, organic acid, food biotechnology, biodegradation, and antibiotics (68) and can convert carbohydrates to organic acids such as acetic acid (69). However, further study is needed to confirm their specific roles in the fermentation of polysaccharides in the intestine.

## Conclusion

We concluded that EP inclusion in chickens' diet improves growth performance, enhances intestinal immune response and integrity, and modulates the caecal microbiota of broilers. This study suggests the application of EP as an alternative to antibiotics in chicken and also provides insight to develop marine algae polysaccharide-based functional food and therapeutic agent.

## Data Availability Statement

The original contributions presented in the study are publicly available. This data can be found here: https://www.ncbi.nlm.nih.gov/bioproject/765947.

## Ethics Statement

The animal study was reviewed and approved by Animal Care and Use Committee of the Institute of Subtropical Agriculture, Chinese Academy of Sciences, Hunan, China.

## Author Contributions

TW: conceptualization, investigation, data generation, curation, and original draft preparation. XD, KG, and ZY: data generation and curation and editing. ZL: methodology, investigation, data generation, curation, and reviewing. YY, CX, and XW: conceptualization, supervision, validation, reviewing, and editing. All authors contributed to the article and approved the submitted version.

## Funding

We would like to acknowledge the earmarked fund for National Natural Science Foundation of China (31902196), China Agriculture Research System (CARS-35), China Postdoctoral Science Foundation-funded project (2021M693383 and 2019M662273), and Taishan industry leading talent blue talent project for their financial support.

## Conflict of Interest

The authors declare that the research was conducted in the absence of any commercial or financial relationships that could be construed as a potential conflict of interest.

## Publisher's Note

All claims expressed in this article are solely those of the authors and do not necessarily represent those of their affiliated organizations, or those of the publisher, the editors and the reviewers. Any product that may be evaluated in this article, or claim that may be made by its manufacturer, is not guaranteed or endorsed by the publisher.

## References

[1] Dibner J Richards J. Antibiotic growth promoters in agriculture: history and mode of action. Poult Sci. (2005) 84:634–43. 10.1093/ps/84.4.634 15844822

[2] Fardet A. New hypotheses for the health-protective mechanisms of whole-grain cereals: what is beyond fibre? Nutr Res Rev. (2010) 23:65–134. 10.1017/S0954422410000041 20565994

[3] Nicholson JK Holmes E Kinross J Burcelin R Gibson G Jia W. Host-gut microbiota metabolic interactions. Science. (2012) 336:1262–7. 10.1126/science.1223813 22674330

[4] Jeffery IB O'Toole PW. Diet-microbiota interactions and their implications for healthy living. Nutrients. (2013) 5:234–52. 10.3390/nu5010234 23344252 PMC3571646

[5] Stanley D Hughes RJ Moore RJ. Microbiota of the chicken gastrointestinal tract: influence on health, productivity and disease. Appl Microbiol Biotechnol. (2014) 98:4301–10. 10.1007/s00253-014-5646-2 24643736

[6] Sergeant MJ Constantinidou C Cogan TA Bedford MR Penn CW Pallen MJ. Extensive microbial and functional diversity within the chicken cecal microbiome. PLoS ONE. (2014) 9:e91941. 10.1371/journal.pone.0091941 24657972 PMC3962364

[7] Mishra SP Karunakar P Taraphder S Yadav H. Free fatty acid receptors 2 and 3 as microbial metabolite sensors to shape host health: pharmacophysiological view. Biomedicines. (2020) 8:154. 10.3390/biomedicines8060154 32521775 PMC7344995

[8] Berg D Clemente JC Colombel JF. Can inflammatory bowel disease be permanently treated with short-term interventions on the microbiome? Expert Rev Gastroenterol Hepatol. (2015) 9:781–95. 10.1586/17474124.2015.1013031 25665875

[9] Zhang Y Duan X Wassie T Wang H Li T Xie C. Enteromorpha prolifera polysaccharide-zinc complex modulates the immune response and alleviates LPS-induced intestinal inflammation via inhibiting the TLR4/NF-κB signaling pathway. Food Funct. (2021). 10.1039/d1fo02171k. [Epub ahead of print]. 34704575

[10] Jiao L Jiang P Zhang L Wu M. Antitumor and immunomodulating activity of polysaccharides from *Enteromorpha intestinalis*. Biotechnol Bioproc Eng. (2010) 15:421–8. 10.1007/s12257-008-0269-z

[11] Wijesekara I Pangestuti R Kim SK. Biological activities and potential health benefits of sulfated polysaccharides derived from marine algae. Carbohydr Polym. (2011) 84:14–21. 10.1016/j.carbpol.2010.10.06234692752

[12] Wassie T Niu K Xie C Wang H Wu X. Extraction techniques, biological activities and health benefits of marine algae *Enteromorpha prolifera* polysaccharide. Front Nutr. (2021) 8:747928. 10.3389/fnut.2021.747928 34692752 PMC8529069

[13] Chi Y Li Y Zhang G Gao Y Ye H Gao J. Effect of extraction techniques on properties of polysaccharides from *Enteromorpha prolifera* and their applicability in iron chelation. Carbohydr Polym. (2018) 181:616–23. 10.1016/j.carbpol.2017.11.104 29254014

[14] Yu Y Li Y Du C Mou H Wang P. Compositional and structural characteristics of sulfated polysaccharide from *Enteromorpha prolifera*. Carbohydr Polym. (2017) 165:221–8. 10.1016/j.carbpol.2017.02.011 28363544

[15] Guo Y Zhao ZH Pan ZY An LL Balasubramanian B Liu WC. New insights into the role of dietary marine-derived polysaccharides on productive performance, egg quality, antioxidant capacity, and jejunal morphology in late-phase laying hens. Poult Sci. (2020) 99:2100–7. 10.1016/j.psj.2019.12.032 32241495 PMC7587743

[16] Liu WC Guo Y Zhihui Z Jha R Balasubramanian B. Algae-derived polysaccharides promote growth performance by improving antioxidant capacity and intestinal barrier function in broiler chickens. Front Vet Sci. (2020) 7:990. 10.3389/fvets.2020.601336 33344535 PMC7738339

[17] Zhao Y Balasubramanian B Guo Y Qiu SJ Jha R Liu WC. Dietary *Enteromorpha* polysaccharides supplementation improves breast muscle yield and is associated with modification of mRNA transcriptome in broiler chickens. Front Vet Sci. (2021) 8:337. 10.3389/fvets.2021.663988 33937385 PMC8085336

[18] Liu WC Zhu YR Zhao ZH Jiang P Yin FQ. Effects of dietary supplementation of algae-derived polysaccharides on morphology, tight junctions, antioxidant capacity and immune response of duodenum in broilers under heat stress. Animals. (2021) 11:2279. 10.3390/ani11082279 34438737 PMC8388401

[19] Qiu SJ Zhang R Guo Y Zhao Y Zhao ZH Liu WC. Transcriptome analysis reveals potential mechanisms of the effects of dietary *Enteromorpha* polysaccharides on bursa of fabricius in broilers. Vet Med Sci. (2021) 7:1881–9. 10.1002/vms3.573 34265184 PMC8464242

[20] Liu WC Zhou SH Balasubramanian B Zeng F-Y Sun CB Pang HY. Dietary seaweed (*Enteromorpha*) polysaccharides improve growth performance involved in the regulation of immune responses, intestinal morphology and microbial community in banana shrimp *Fenneropenaeus merguiensis*. Fish Shellfish Immunol. (2020) 104:202–12. 10.1016/j.fsi.2020.05.079 32504803

[21] Zhang Z Wang X Han S Liu C Liu F. Effect of two seaweed polysaccharides on intestinal microbiota in mice evaluated by illumina PE250 sequencing. Int J Biol Macromol. (2018) 112:796–802. 10.1016/j.ijbiomac.2018.01.192 29427682

[22] Kong Q Dong S Gao J Jiang C. In vitro fermentation of sulfated polysaccharides from *E. prolifera* and *L. japonica* by human fecal microbiota. Int J Biol Macromol. (2016) 91:867–71. 10.1016/j.ijbiomac.2016.06.036 27316763

[23] Xie C Zhang Y Niu K Liang X Wang H Shan J. *Enteromorpha* polysaccharide-Zinc replacing prophylactic antibiotics contributes to improving gut health of weaned piglets. Anim Nutr. (2021) 7:641–9. 10.1016/j.aninu.2021.01.008 34401542 PMC8340054

[24] Guo Y Balasubramanian B Zhao ZH Liu WC. Marine algal polysaccharides alleviate aflatoxin B1-induced bursa of fabricius injury by regulating redox and apoptotic signaling pathway in broilers. Poult Sci. (2021) 100:844–57. 10.1016/j.psj.2020.10.050 33518138 PMC7858151

[25] Lv H Xiao B Gao Y. Study on the extraction, purification and structural characterization of polysaccharide from *Enteromorpha*. Food Res Dev. (2013) 34:33–6.

[26] Aviagen R. Ross Broiler Management Manual, 2009. (2014). Available online at: http://ptaviagen.com/assets/Tech_Center/Ross_broiler/Ross_broiler_manual_

[27] Shang HM Song H Xing YL Niu SL Ding GD Jiang YY. Effects of dietary fermentation concentrate of *Hericium caput-medusae* (bull: Fr.) Pers. on growth performance, digestibility, and intestinal microbiology and morphology in broiler chickens. J Sci Food Agric. (2016) 96:215–22. 10.1002/jsfa.7084 25582752

[28] Livak KJ Schmittgen TD. Analysis of relative gene expression data using real-time quantitative PCR and the 2^−ΔΔCT^ method. Methods. (2001) 25:402–8. 10.1006/meth.2001.1262 11846609

[29] Saarinen MT Kärkkäinen O Hanhineva K Tiihonen K Hibberd A Mäkelä KA. Metabolomics analysis of plasma and adipose tissue samples from mice orally administered with polydextrose and correlations with cecal microbiota. Sci Rep. (2020) 10:21577. 10.1038/s41598-020-78484-y 33299048 PMC7726573

[30] Yang S Shan C Ma X Qin Y Ju A Duan A. Immunomodulatory effect of Acanthopanax senticosus polysaccharide on immunosuppressed chickens. Poult Sci. (2021) 100:623–30. 10.1016/j.psj.2020.11.059 33518115 PMC7858182

[31] Liu Y Wu X Jin W Guo Y. Immunomodulatory effects of a low-molecular-weight polysaccharide from *Enteromorpha prolifera* on RAW 264.7 macrophages and cyclophosphamide-induced immunosuppression mouse models. Mar Drugs. (2020) 18:340. 10.3390/md18070340 32605327 PMC7401259

[32] Zhou Z Pan S Wu S. Modulation of the growth performance, body composition and nonspecific immunity of crucian carp *Carassius auratus* upon *Enteromorpha prolifera* polysaccharide. Int J Biol Macromol. (2020) 147:29–33. 10.1016/j.ijbiomac.2020.01.065 31923485

[33] Wei J Wang S Liu G Pei D Liu Y Liu Y. Polysaccharides from *Enteromorpha prolifera* enhance the immunity of normal mice. Int J Biol Macromol. (2014) 64:1–5. 10.1016/j.ijbiomac.2013.11.013 24296406

[34] Kim J-K Cho ML Karnjanapratum S Shin I-S You SG. In vitro and in vivo immunomodulatory activity of sulfated polysaccharides from *Enteromorpha prolifera*. Int J Biol Macromol. (2011) 49:1051–8. 10.1016/j.ijbiomac.2011.08.032 21907732

[35] Liu WC Ou BH Liang ZL Zhang R Zhao ZH. Algae-derived polysaccharides supplementation ameliorates heat stress-induced impairment of bursa of fabricius via modulating NF-κB signaling pathway in broilers. Poult Sci. (2021) 100:101139. 10.1016/j.psj.2021.101139 34225200 PMC8264154

[36] Burns K Martinon F Esslinger C Pahl H Schneider P Bodmer JL. MyD88, an adapter protein involved in interleukin-1 signaling. J Biol Chem. (1998) 273:12203–9. 10.1074/jbc.273.20.12203 9575168

[37] Janssens S Burns K Vercammen E Tschopp J Beyaert R. MyD88S, a splice variant of MyD88, differentially modulates NF-κB-and AP-1-dependent gene expression. FEBS Lett. (2003) 548:103–7. 10.1016/S0014-5793(03)00747-6 12885415

[38] Yamamoto M Sato S Hemmi H Hoshino K Kaisho T Sanjo H. Role of adaptor TRIF in the MyD88-independent toll-like receptor signaling pathway. Science. (2003) 301:640–3. 10.1126/science.1087262 12855817

[39] Deguine J Barton GM. MyD88: a central player in innate immune signaling. F1000Prime Rep. (2014) 6:97. 10.12703/P6-97 25580251 PMC4229726

[40] Wang Z Zhang Z Zhao J Yong C Mao Y. Polysaccharides from *Enteromorpha prolifera* ameliorate acute myocardial infarction *in vitro* and i*n vivo* via up-regulating HIF-1α. Int Heart J. (2019) 60:964–73. 10.1536/ihj.18-519 31257333

[41] Caspary WF. Physiology and pathophysiology of intestinal absorption. Am J Clin Nutr. (1992) 55:299S−308S. 10.1093/ajcn/55.1.299s 1728844

[42] Onderci M Sahin N Sahin K Cikim G Aydin A Ozercan I. Efficacy of supplementation of α-amylase-producing bacterial culture on the performance, nutrient use, and gut morphology of broiler chickens fed a corn-based diet. Poult Sci. (2006) 85:505–10. 10.1093/ps/85.3.505 16553283

[43] Silva MAd Pessotti BMdS Zanini SF Colnago GL Rodrigues MRA Nunes LdC. Intestinal mucosa structure of broiler chickens infected experimentally with *Eimeria tenella* and treated with essential oil of oregano. Ciencia Rural. (2009) 39:1471–7. 10.1590/S0103-84782009005000135

[44] Ma N Tian Y Wu Y Ma X. Contributions of the interaction between dietary protein and gut microbiota to intestinal health. Curr Protein Peptide Sci. (2017) 18:795–808. 10.2174/1389203718666170216153505 28215168

[45] Liu T Li J Liu Y Xiao N Suo H Xie K. Short-chain fatty acids suppress lipopolysaccharide-induced production of nitric oxide and proinflammatory cytokines through inhibition of NF-κB pathway in RAW264. 7 cells. Inflammation. (2012) 35:1676–84. 10.1007/s10753-012-9484-z 22669487

[46] Yu Y Sitaraman S Gewirtz AT. Intestinal epithelial cell regulation of mucosal inflammation. Immunol Res. (2004) 29:55–67. 10.1385/IR:29:1-3:055 15181270

[47] Al-Sadi R Khatib K Guo S Ye D Youssef M Ma T. Occludin regulates macromolecule flux across the intestinal epithelial tight junction barrier. Am J Physiol Gastrointest Liver Physiol. (2011) 300:G1054–64. 10.1152/ajpgi.00055.2011 21415414 PMC3119114

[48] Fujibe M Chiba H Kojima T Soma T Wada T Yamashita T. Thr203 of claudin-1, a putative phosphorylation site for MAP kinase, is required to promote the barrier function of tight junctions. Exp Cell Res. (2004) 295:36–47. 10.1016/j.yexcr.2003.12.014 15051488

[49] Erturk-Hasdemir D Oh SF Okan NA Stefanetti G Gazzaniga FS Seeberger PH. Symbionts exploit complex signaling to educate the immune system. Proc Natl Acad Sci USA. (2019) 116:26157–66. 10.1073/pnas.1915978116 31811024 PMC6936714

[50] Birchenough GM Nyström EE Johansson ME Hansson GC. A sentinel goblet cell guards the colonic crypt by triggering Nlrp6-dependent Muc2 secretion. Science. (2016) 352:1535–42. 10.1126/science.aaf7419 27339979 PMC5148821

[51] Higuchi M Matsuo A Shingai M Shida K Ishii A Funami K. Combinational recognition of bacterial lipoproteins and peptidoglycan by chicken Toll-like receptor 2 subfamily. Dev Comparat Immunol. (2008) 32:147–55. 10.1016/j.dci.2007.05.003 17614130

[52] Chen JQ Szodoray P Zeher M. Toll-like receptor pathways in autoimmune diseases. Clin Rev Allergy Immunol. (2016) 50:1–17. 10.1007/s12016-015-8473-z 25687121

[53] Tan J McKenzie C Potamitis M Thorburn AN Mackay CR Macia L. The role of short-chain fatty acids in health and disease. Adv Immunol. (2014) 121:91–119. 10.1016/B978-0-12-800100-4.00003-9 24388214

[54] Shi LZ Wang R Huang G Vogel P Neale G Green DR. HIF1α-dependent glycolytic pathway orchestrates a metabolic checkpoint for the differentiation of TH17 and Treg cells. J Exp Med. (2011) 208:1367–76. 10.1084/jem.20110278 21708926 PMC3135370

[55] Everts B Amiel E Huang SCC Smith AM Chang CH Lam WY. TLR-driven early glycolytic reprogramming via the kinases TBK1-IKKε supports the anabolic demands of dendritic cell activation. Nat Immunol. (2014) 15:323–32. 10.1038/ni.2833 24562310 PMC4358322

[56] Zhang W Zhang X Zou K Xie J Zhao S Liu J. Seabuckthorn berry polysaccharide protects against carbon tetrachloride-induced hepatotoxicity in mice via anti-oxidative and anti-inflammatory activities. Food Funct. (2017) 8:3130–8. 10.1039/C7FO00399D 28766672

[57] Gobet A Mest L Perennou M Dittami SM Caralp C Coulombet C. Seasonal and algal diet-driven patterns of the digestive microbiota of the European abalone *Haliotis tuberculata*, a generalist marine herbivore. Microbiome. (2018) 6:60. 10.1186/s40168-018-0430-7 29587830 PMC5870069

[58] Hills RD Pontefract BA Mishcon HR Black CA Sutton SC Theberge CR. Gut microbiome: profound implications for diet and disease. Nutrients. (2019) 11:1613. 10.3390/nu11071613 31315227 PMC6682904

[59] Kartzinel TR Hsing JC Musili PM Brown BR Pringle RM. Covariation of diet and gut microbiome in African megafauna. Proc Natl Acad Sci USA. (2019) 116:23588–93. 10.1073/pnas.1905666116 31685619 PMC6876249

[60] Bäckhed F Ding H Wang T Hooper LV Koh GY Nagy A. The gut microbiota as an environmental factor that regulates fat storage. PNAS. (2004) 101:15718–23. 10.1073/pnas.0407076101 15505215 PMC524219

[61] Flint HJ Scott KP Duncan SH Louis P Forano E. Microbial degradation of complex carbohydrates in the gut. Gut Microbes. (2012) 3:289–306. 10.4161/gmic.19897 22572875 PMC3463488

[62] Xu J Mahowald MA Ley RE Lozupone CA Hamady M Martens EC. Evolution of symbiotic bacteria in the distal human intestine. PLoS Biol. (2007) 5:e156. 10.1371/journal.pbio.0050156 17579514 PMC1892571

[63] Hooper LV. Bacterial contributions to mammalian gut development. Trends Microbiol. (2004) 12:129–34. 10.1016/j.tim.2004.01.001 15001189

[64] Sears CL. A dynamic partnership: celebrating our gut flora. Anaerobe. (2005) 11:247–51. 10.1016/j.anaerobe.2005.05.001 16701579

[65] Xu Y Li J Han X Zhang Z Zhong M Hu Z. *Enteromorpha prolifera* diet drives intestinal microbiome composition in *Siganus oramin*. Curr Microbiol. (2020) 78:229–37. 10.1007/s00284-020-02218-6 33034768

[66] Rey MW Ramaiya P Nelson BA Brody KSD Zaretsky EJ Tang M. Complete genome sequence of the industrial bacterium *Bacillus licheniformis* and comparisons with closely related bacillus species. Genome Biol. (2004) 5:R77. 10.1186/gb-2004-5-10-r77 15461803 PMC545597

[67] Lee C Kim JY Song HS Kim YB Choi YE Yoon C. Genomic analysis of *Bacillus licheniformis* CBA7126 isolated from a human fecal sample. Front Pharmacol. (2017) 8:724. 10.3389/fphar.2017.00724 29081747 PMC5645497

[68] Shameer S. Haloalkaliphilic Bacillus species from solar salterns: an ideal prokaryote for bioprospecting studies. Ann Microbiol. (2016) 66:1315–27. 10.1007/s13213-016-1221-7

[69] Paavilainen S Helistö P Korpela T. Conversion of carbohydrates to organic acids by *Alkaliphilic bacilli*. J Ferment Bioeng. (1994) 78:217–22. 10.1016/0922-338X(94)90293-3

